# A method for inverse bifurcation of biochemical switches: inferring parameters from dose response curves

**DOI:** 10.1186/s12918-014-0114-2

**Published:** 2014-11-20

**Authors:** Irene Otero-Muras, Pencho Yordanov, Joerg Stelling

**Affiliations:** BioProcess Engineering Group, IIM-CSIC (Spanish Council for Scientific Research), Eduardo Cabello 6, Vigo, 36208 Spain; Department of Biosystems Science and Engineering, Swiss Federal Institute of Technology (ETH Zurich), Universitatstrasse 19, 8092 Zurich, Switzerland

**Keywords:** Biochemical reaction network, Bistability, Saddle node bifurcation, Dose response curve, Chemical reaction network theory

## Abstract

**Background:**

Within cells, stimuli are transduced into cell responses by complex networks of biochemical reactions. In many cell decision processes the underlying networks behave as bistable switches, converting graded stimuli or inputs into all or none cell responses. Observing how systems respond to different perturbations, insight can be gained into the underlying molecular mechanisms by developing mathematical models. Emergent properties of systems, like bistability, can be exploited to this purpose. One of the main challenges in modeling intracellular processes, from signaling pathways to gene regulatory networks, is to deal with high structural and parametric uncertainty, due to the complexity of the systems and the difficulty to obtain experimental measurements. Formal methods that exploit structural properties of networks for parameter estimation can help to overcome these problems.

**Results:**

We here propose a novel method to infer the kinetic parameters of bistable biochemical network models. Bistable systems typically show hysteretic dose response curves, in which the so called bifurcation points can be located experimentally. We exploit the fact that, at the bifurcation points, a condition for multistationarity derived in the context of the Chemical Reaction Network Theory must be fulfilled. Chemical Reaction Network Theory has attracted attention from the (systems) biology community since it connects the structure of biochemical reaction networks to qualitative properties of the corresponding model of ordinary differential equations. The inverse bifurcation method developed here allows determining the parameters that produce the expected behavior of the dose response curves and, in particular, the observed location of the bifurcation points given by experimental data.

**Conclusions:**

Our inverse bifurcation method exploits inherent structural properties of bistable switches in order to estimate kinetic parameters of bistable biochemical networks, opening a promising route for developments in Chemical Reaction Network Theory towards kinetic model identification.

**Electronic supplementary material:**

The online version of this article (doi:10.1186/s12918-014-0114-2) contains supplementary material, which is available to authorized users.

## Background

In the context of systems biology, modeling is used to drive the acquisition of knowledge about the molecular mechanisms governing intracellular processes by evaluating the system’s behavior. In this way, measuring how the levels of some key species in the network evolve, for example, upon environmental perturbations, may help to retrieve important features concerning connectivities and rate constants of the underlying mechanism (i.e. model structure and parameters, in mathematical modeling terms). One key challenge in systems biology is the identification of kinetic models from limited quantitative data [1,2], hence the increasing interest in results connecting structure, parameters and dynamic behavior [3-5].

Biochemical switches are biochemical reaction networks with the capacity for two different stable steady states. Depending on the initial and environmental conditions the system will evolve towards one steady state or the other. This property enables the cell to *take decisions*, by transforming, for example, graded stimuli into all or none responses. Bistable switches are ubiquitous in cell signaling networks, from pheromone sensing [6] to cell proliferation regulation [7]. The presence of bistability reveals structural properties that the network might or might not have, allowing to discriminate among different structural hypotheses. There are a number of results connecting particular structural features with the absence or presence of bistability in biochemical reaction networks. Some of these results are central to the Chemical Reaction Network Theory (CRNT) [8,9], which has received in the recent years a considerable amount of attention from the (systems) biology community [10].

How can bistability bring insight on the parameters of a model?

The *forward problem* of determining the mapping from the parameter space to the space of the model behavior and, in particular, to the capacity of the model for bistability, can be approached using standard bifurcation analysis [11] when the number of parameters is small. To build bifurcation diagrams the steady states of the system are computed by varying one or a small number of parameters with the rest of the parameters being fixed. In a previous work [12] a condition for multiple steady states was derived within the framework of CRNT. The condition can be easily checked by evaluating the determinant of a matrix, thus providing an efficient tool to tackle the aforementioned forward problem: once values are assigned to the parameters it is immediate to assess the capacity or not for multiple steady states.

The inverse problem of finding parameter regions compatible with bistable behaviour can be tackled by checking the multistationarity condition derived in [12] through the whole parameter space. In [12] an algorithm based on interval methods was suggested for a reliable and systematic search in order to obtain a complete partition of the parameter space. Starting from a reaction mechanism with (qualitative) experimental evidence of bistability, the algorithm allowed us, based on this qualitative information, to restrict the parameter space to where multiple steady states can be found.

Here, our goal is to exploit quantitative information from experimental data in order to infer the actual values of the model parameters, and what we develop is a method for parameter estimation from experimental data. Typical evidences for bistability obtained from experiments are, for example, hysteretic dose response curves. Dose response curves indicate how the steady state of a system evolves upon increase or decrease of a given stimulus. In a hysteretic dose response curve a continuous change in the stimulus (dose or input), produces a discontinuous jump in the measured steady state concentration or output beyond the so called bifurcation point. In this work we address the inverse problem of determining the parameters that produce certain desired properties of the bifurcation diagram and, consequently, in the observed experimental dose response curves. In particular, the method uses the location of the bifurcation points given by experimental data^a^ to infer the kinetic parameters, exploiting the condition derived in [12]. In contrast to other methods developed for inverse bifurcation analysis which use generic bifurcation conditions [13,14] and can therefore be used for any system of ordinary differential equations, our method is specific for chemical reaction networks since it exploits their particular structural properties, bringing additional insight into the inverse bifurcation problem for chemical reaction systems.

The basic ingredients of the inverse bifurcation method are described in the Methods section, where a brief introduction of some essential CRNT concepts is given together with the condition for multiple steady states derived in [12], here extended to a broader class of systems. The main contribution of the paper is presented in the Results and discussion section where we develop the inverse bifurcation method exploiting the multistationarity condition to infer kinetic parameters of bistable networks. The method presented provides an alternative way of estimating parameters with few (but very informative) experimental data, and can also be combined with standard techniques (estimation from time course measurements etc). As a proof of concept we apply our method to estimate the kinetic parameters of a genetic toggle switch model.

## Methods

### Reaction network graph and dynamics

We consider biochemical reaction networks with mass action kinetics where the dynamics are modeled by systems of ordinary differential equations (ODEs). The structure of mass action kinetic models is given by the connectivity and the stoichiometry of the network and the parameters are the kinetic rate constants. Let us denote by *m* the number of species and by *r* the number of reactions in a reaction network, the balance equations describing the dynamics of the concentrations of the species involved are a set of ODEs usually written as $\dot {c} = N v(c)$, with $c \in \mathbb {R}^{m}_{\geq 0}$ being the vector of concentrations, $N \in \mathbb {R}^{m \times r}$ the stoichiometric matrix and $v \in \mathbb {R}^{r}$ the vector of reaction rates^b^. Within the context of CRNT alternative ways to encode the balance equations are used such that the connection between structure and dynamics can be more easily exploited [4]. Results in this paper rely on a particular representation of the balance equations already introduced in [8], which is based on the graph of complexes of a reaction network and briefly presented next.

**Figure 1:**
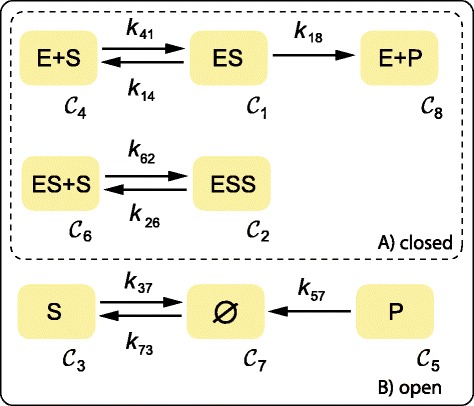
**Graph of complexes for the protein activation mechanism.** Every node of the graph corresponds to a network complex. The zero complex (*∅*) represents the environment.

For illustrative purposes we consider the mechanism in Figure 1 where the transformation of a protein *S* to its active form *P* is mediated by another protein or enzyme *E*, whereas *S* inhibits its own activation. The complexes of the network are the linear combinations of species at both sides of the reaction arrows and the number of complexes is denoted by *n*. In the *graph of complexes* of a reaction network the nodes are the complexes $\mathcal {C}_{1}, \hdots,\mathcal {C}_{n}$ and the edges are the reactions. The subgraphs are called *linkage classes* and the number of linkage classes is denoted by *ℓ*. Two nodes $\mathcal {C}_{i}$, $\mathcal {C}_{j}$ are *strongly linked* if there is a directed path from $\mathcal {C}_{i}$ to $\mathcal {C}_{j}$ and also a directed path from $\mathcal {C}_{j}$ to $\mathcal {C}_{i}$. A *terminal strong linkage class* is a maximal set of nodes within a linkage class such that there is no edge pointing to any other set of nodes strongly linked. The number of terminal linkage classes is denoted by *t*. Networks with *t*>*ℓ* are considered in general to be unsuited for the description of real chemical systems [8]. In what follows, we focus on networks where *t*=*ℓ* (note that this includes weakly reversible networks, i.e. networks where every linkage class is a strong terminal linkage class).

For a reaction network being bistable there is necessarily an exchange of matter and/or energy through the boundary of the system. According to the second law of thermodynamics isolated systems evolve towards the thermodynamic equilibrium. Isolated reaction networks endowed with mass action kinetics achieve the thermodynamic equilibrium which has been demonstrated to be globally asymptotically stable [15,16] precluding any kind of complex nonlinear behavior such as bistability or limit cycles. A reaction kinetic system is thermodynamically isolated if there is no matter exchange with the environment and the kinetic constants are such that the detailed balance condition is fulfilled [9].

Exchange of matter can appear implicitly, as it happens in reactions of the form *S*→2*S*, or explicitly in the form of pseudo reactions of the species that are entering or going out of the control volume with a zero complex representing the environment. In the context of signaling, we use the same formalism of pseudo reactions with the environment to account for the formation or degradation of a species *S* (*∅*→*S* or *S*→*∅* respectively).

In our example we consider the degradation of the species *S* and *P*, and the constitutive formation of the inactive form *S*. The corresponding graph of complexes is depicted in Figure 1 where every complex is labeled with a number *i* from 1 to *n*.

Once species and complexes are labeled (note that the ordering of both the species and the complexes is arbitrary) we build the molecularity matrix $Y \in \mathbb {R}^{m \times n}$, where *y*_*ij*_ is the molecularity of the species *i* in the complex $\mathcal {C}_{j}$. Every complex $\mathcal {C}_{j}$ for *j*=1,…,*n* has an associated mass action monomial:

(1) $$ \psi_{j} (c) = \prod_{i=1}^{m} c_{i}^{y_{ij}},  $$

and every arrow in the graph, going from complex $\mathcal {C}_{j}$ to complex $\mathcal {C}_{k}$ is weighted by a rate constant *k*_*jk*_. The corresponding reaction rate is *v*_*jk*_(*c*)=*k*_*jk*_*ψ*_*j*_(*c*). Within this framework the balance equations read:

(2) $$ \dot{c} = Y A \psi (c),  $$

where the matrix $A\in \mathbb {R}^{n \times n}$ is built from the adjacency matrix of the graph and contains the kinetic rate constants [8].

As a consequence of the mass conservation laws the trajectories of Eq. (2) in the concentration space are constrained to a convex region known as the reaction polyhedron [17] or *stoichiometric compatibility class*, which is next defined after introducing the *stoichiometric subspace*. The *stoichiometric subspace* is the subspace spanned by the columns of the stoichiometric matrix *N*. Considering the formulation in Eq. (2), it can be defined for networks with *t*=*ℓ* as $\mathcal {S}:=Im(YA)$. We denote by *s* the dimension of the stoichiometric subspace, i.e. $dim(\mathcal {S})=s$. The number of independent conservation relations is given by *m*−*s*. Let *B* be a matrix such that the rows of *B*^*T*^ are a basis of the left null space^c^ of *YA*, i. e. (*YA*)^*T*^*B*=0. Let us take a reference concentration vector *c*_0_ and define

(3) $$ W(c;c_{0}) = B^{T} (c-c_{0}),  $$

the *reaction polyhedron* consists of all *c*≥0 such that *W*(*c*;*c*_0_)=0. Closed reaction networks are conservative systems [9], i.e. all the species participate in at least one conservation law and therefore there is a strictly positive row vector *v* (with *m* elements) such that *vS*=0. On the contrary, open systems are not conservative. The existence of conservation laws implies that the ODE system (2) is not minimal, i.e. some equations depend linearly on the others. We can alternatively describe the system by a set of *s* linearly independent ODEs and a set of *m*−*s* algebraic equations of the form:

(4) $$ B^{T} c = b,  $$

with *b*=*B*^*T*^*c*_0_. The original system of ODEs is thus transformed into a system of differential algebraic equations (DAEs). The derivation of the set of ODEs (2) and the DAE system for the protein activation network is included in Additional file 1.

### Locus of equilibria and tangent bifurcation condition

A condition for multiple steady states in biochemical reaction networks is derived and demonstrated in [12] for weakly reversible networks. In the context of intracellular processes, networks are often non weakly reversible. However, in the general case they fulfill *t*=*ℓ*, i.e. they have a number of linkage classes equal to the number of terminal linkage classes (as indicated previously, networks with *t*>*ℓ* might not be suitable to represent realistic systems). For networks with *t*=*ℓ* the condition in [12] is still valid. For the sake of completeness, we briefly summarize next the derivation of this condition, simplified and adapted to *t*=*ℓ* networks. The reader can find the detailed derivation and proof in [12], from which the validity for *t*=*ℓ* networks follows.

The system in Eq. (2) is at equilibrium for any *c* that satisfies *YAψ*(*c*)=0. Equivalently, one can say that a vector $z\in \mathbb {R}^{n}$ defined as *z*=*Aψ*(*c*) belongs to the *deficiency subspace*:

(5) $$ D_{\delta}:=Ker(Y) \bigcap Im(A).  $$

The dimension of this subspace is the *deficiency* of the network, *δ*=*dim*(*D*_*δ*_). For networks with *t*=*ℓ* the deficiency is given by the formula *δ*=*n*−*ℓ*−*s*, and a basis *ω*_1_,…,*ω*_*δ*_ for the deficiency subspace *D*_*δ*_ can be computed from graph matrices taking into account that:

(6) $$ span(\omega) = Ker \left[\begin{array}{c} Y \\ \Lambda^{T}\end{array} \right],  $$

where *Λ* is a *n*×*ℓ* matrix in which entry *Λ*_*ij*_= corresponds to the node $\mathcal {C}_{i}$ in the linkage class $\mathcal {L}_{j}$ such that *Λ*_*ij*_=1 if $\mathcal {C}_{i} \in \mathcal {L}_{j}$ and *Λ*_*ij*_=0 otherwise.

Since the vector *z* defined above is a linear combination of the vectors of the basis of *D*_*δ*_, we have:

(7) $$ A\psi(c)=\sum_{i=1}^{\delta} \alpha_{i} \omega_{i}.  $$

On the other hand, from Eq. (1) provided that *ψ*(*c*)>0, we get:

(8) $$ \ln \psi(c) = Y \ln c.  $$

From Eqs. (7) and (8), a set of *n*−*ℓ* linearly independent equations can be obtained in terms of the state vector $c \in \mathbb {R}_{> 0}^{m}$, the deficiency parameter vector $\alpha \in \mathbb {R}^{\delta }$ and the kinetic parameter vector $k \in \mathbb {R}_{>0}^{r}$:

(9) $$ \mathcal{H}_{s}(c,\alpha;k)=0,  $$

where $\mathcal {H}_{s}(c,\alpha ;k)\!: \mathbb {R}^{m}_{>0} \times \mathbb {R}^{\delta } \rightarrow \! \mathbb {R}^{n-\ell }$ is continuous and differentiable, given a rate constant vector *k*. Eq. (9) describes the locus of equilibria of a reaction network (see Additional file 1 for the detailed derivation of this expression in the protein activation network example). Wherever this locus is continuous Eq. (9) defines the so called *equilibrium manifold* of dimension *λ*=*m*−*s*.

**Figure 2:**
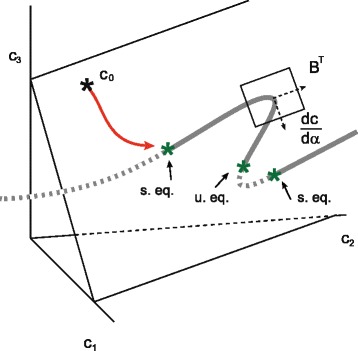
**Scheme illustrating the condition for multistationarity for** ***λ*** **=** ***δ*** **=1.** In this diagram the intersections between the equilibrium manifold and the reaction polyhedron correspond to two stable equilibrium points (s. eq.) and one unstable equilibrium point (u. eq.).

The system in Eq. (2) together with an initial concentration vector *c*_0_ describes an initial value problem. A reaction network has a unique steady state if for all the initial conditions stoichiometrically compatible with *c*_0_ the system reaches the same equilibrium *c*^∗^, which is also stoichiometrically compatible with *c*_0_. The steady state is, in fact, the intersection between the equilibrium manifold and the reaction polyhedron defined by *W*(*c*;*c*_0_)=0, where *W* has been introduced in Eq. (3). Typical bistable systems show three intersections, one of them corresponding to an unstable steady state [18], as illustrated in Figure 2. If the equilibrium manifold and the reaction polyhedron become tangent at some point of the state space, multiple steady states appear, at least, for some stoichiometric compatibility classes. Here it is important to remind that if the network has deficiency 0 and is weakly reversible then bistability is necessarily excluded according to the deficiency zero theorem [8].

Let us assume *δ*=*λ*=1 such that the manifold is a one dimensional curve. Starting from the equilibrium manifold (9) and taking derivatives with respect to *α*, we obtain^d^$D_{c} \mathcal {H}_{s}\frac {dc}{d\alpha }(\alpha ; k)+D_{\alpha } \mathcal {H}_{s}=0$ and therefore the derivative of *c* with respect to *α* is given by

(10) $$ \frac{dc}{d\alpha}\left(\alpha; k\right)=-D_{c} \mathcal{H}_{s}^{-1} D_{\alpha} \mathcal{H}_{s}.  $$

The equilibrium manifold and the reaction polyhedron are tangent provided that $B^{T} \frac {dc}{d \alpha }(\alpha ; k) = 0$, with *B* being introduced in Eq. (3) (see Figure 2). This geometric condition is generalized for arbitrary deficiency and manifold dimension by defining the following matrix:

(11) $$ G(c,\alpha; k)=\left(\begin{array}{cc} D_{c}\mathcal{H}_{s} & D_{\alpha}\mathcal{H}_{s} \\ D_{c} W & D_{\alpha} W\\ \end{array} \right),  $$

where *D*_*c*_*W*=*B*^*T*^ and *D*_*α*_*W*=0. The equilibrium manifold and the reaction polyhedron become tangent when the determinant of matrix (11) vanishes, i.e.:

(12) $$ det\left(G(c,\alpha; k)\right)=0.  $$

**Figure 3:**
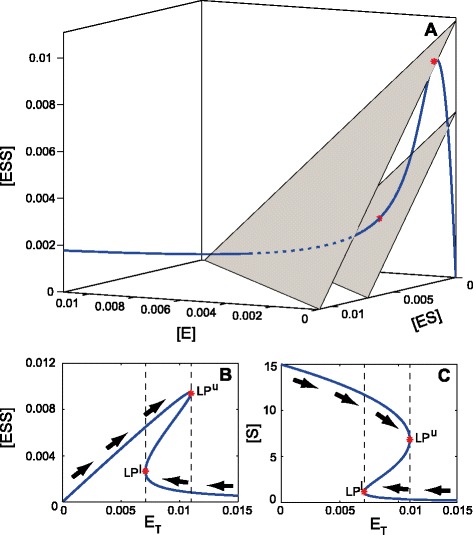
**Equilibrium manifold and tangent bifurcation points for the protein activation mechanism.** **A)** Equilibrium manifold in the space of the species. **B-C)** Bifurcation diagrams showing the steady state concentration of the species ESS and S versus the total enzyme.

The tangent bifurcation condition for the protein activation network is derived in Additional file 1. This network fulfills *δ*=*λ*=1. The corresponding equilibrium curves for a given set of parameters are depicted in Figure 3 where it can be observed how the equilibrium manifold is tangent to the direction of the reaction simplex in two different points of the state space (Figure 3A). These points correspond to tangent bifurcations, also known as saddle nodes or limit point bifurcations, indicated by LP in Figure 3B and C, where the steady state concentration values of the species *ESS* and *S* are depicted with respect to the concentration of total enzyme *E*_*T*_.

## Results and discussion

### Parameter inference from dose response curves: deficiency one networks

*Dose response curves*, also known as stimulus response curves [3], are x-y diagrams commonly used to represent the input-output behavior of biochemical networks where *x* is the magnitude of a measurable stimulus (dose or exposure level) and *y* is the magnitude of the associated system’s response.

In what follows we refer to dose response diagrams obtained when the steady state concentration of a given species (output or *observable*) is plotted against some parameter (input). The parameter can be a kinetic rate or a conservation law constant, i. e. the total amount of an enzyme or conserved moiety, *b*_*i*_ in Eq. (4). Linear combinations of species of the form *q*_*i*_=*Q*_*i*_*c* are also admitted as outputs, where *Q* is a matrix defining a linear map from the space of the species to the space of the *observables*.

**Figure 4:**
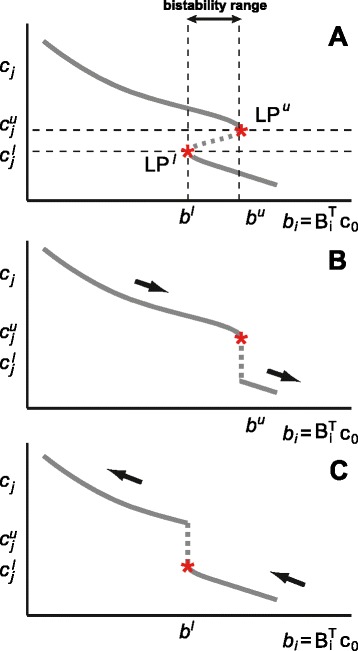
**Scheme of a bifurcation diagram for a bistable reaction network and its corresponding forward and backward dose response curves.** **A)** Bifurcation diagram where the x-axis is the total concentration of a particular moiety and the y-axis is the concentration of a given species. Stars indicate tangent bifurcations also known as saddle nodes or limit points (LP). Unstable equilibria lie on the dashed curve. **B)** Corresponding forward dose response curve. **C)** Corresponding backward dose response curve.

If the structure and parameters of a biochemical reaction network model are known, dose response curves can be obtained computationally from the model equations using a continuation algorithm [19]. In a typical bistable system two tangent bifurcations appear during the equilibrium continuation, where the direction of the parameter reverses as the curve is followed (see Figure 4A). These two tangent bifurcations enclose the range of the bifurcation parameter leading to bistability [18].

Experimentally, dose response curves are obtained by measuring the steady state concentration of a given species for increasing/decreasing values of the parameter at hand. Increasing or decreasing the parameter we obtain the forward (see Figure 4B) or the backward (see Figure 4C) dose response curve respectively. Typical bistable systems show hysteretic behavior, leading to differences between parameter increase (forward curve) and decrease (backward curve) as illustrated in Figure 4B and C. For bistable systems, the tangent bifurcation points in the dose response curves can be located by means of experimental measurements. Details on the experimental procedures are out of the scope of this paper.

Suppose that we know the structure of a biochemical network with capacity for bistability and we want to know the values of the kinetic parameters. Next, we introduce a methodology to infer the parameters of the network from dose response curves, or more precisely, from the *tangent bifurcation points*.

For illustrative purposes we focus first on structures where *δ* = *λ*=1 and we consider dose response curves where the input is the total amount of a particular moiety, i.e. where a conservation law constant *b*_*i*_ is plotted on the x-axis, and the concentration of a species *c*_*j*_, or a combination of species *q*_*j*_, is plotted on the y-axis. As it is illustrated in Figure 4, from the forward and backward dose response curves we can elucidate the values of the parameter *b* where the tangent bifurcations appear, or in other words, the lower and upper values of the mass conservation constant (*b*^*l*^ and *b*^*u*^, respectively) enclosing the region where bistable behavior is observed.

The two points defining the lower and upper bound of the region of bistability in the dose response curve indicate two different stoichiometric compatibility classes tangent to the equilibrium manifold. They correspond, in fact, to two different points in the state space *c*^*l*^=*c*(*α*^*l*^;*k*) and *c*^*u*^=*c*(*α*^*u*^;*k*) where the tangent bifurcation condition is fulfilled. Therefore, we have:

$$B^{T} ~\frac{dc}{d\alpha}\left(\alpha^{l}; k\right) = B^{T} ~\frac{dc}{d\alpha}\left(\alpha^{u}; k\right) = 0,$$

where $\frac {dc}{d\alpha }(\alpha ; k)$ is given by Eq. (10) and *c*(*α*^*l*^;*k*)>0, *c*(*α*^*u*^;*k*)>0 belong to the equilibrium manifold, i.e.:

$$\mathcal{H}_{s}\left(c\left(\alpha^{l}; k\right), \alpha^{l}; k\right)= \mathcal{H}_{s}\left(c\left(\alpha^{u}; k\right), \alpha^{u}; k\right)=0.$$

We search for a parameter vector *k* such that the equilibrium manifold is tangent to the reaction polyhedrons fixed by *b*^*l*^ and *b*^*u*^.

In order to efficiently search for a point in the space of the kinetic parameters satisfying all the conditions above we can solve an optimization problem. First, we define the objective function to be minimized (in this expression *α*^1^=*α*^*l*^, *α*^2^=*α*^*u*^):

$${} J_{0} \left(k, \alpha^{1}, \alpha^{2}\right) = \sum_{i=1}^{2} \left(B^{T} ~\frac{dc}{d\alpha}\left(\alpha^{i}; k\right)\right) ~ \left(\frac{dc}{d\alpha}^{T}\left(\alpha^{i}; k\right) ~B\right), $$

such that at the optimum *J*_0_=0 the geometric condition is fulfilled. The optimization problem can be formulated as:

(13) $${} \begin{aligned} &\text{Minimize} &{}\quad\qquad & J_{0} \left(k, \alpha^{l}, \alpha^{u}\right)~~~~~\left(k \in \mathbb{R}_{>0}^{r}, \alpha^{l}, \alpha^{u} \in \mathbb{R}\right)\\ &\text{subject to:} & \qquad &\mathcal{H}_{s}\left(c^{l}, \alpha^{l}; k\right)=0, ~~\mathcal{H}_{s}\left(c^{u}, \alpha^{u}; k\right)=0 \\ &~&&~~ B^{T} \cdot c^{l} = b^{l}, ~~B^{T} \cdot c^{u} = b^{u} \\ &~&&~~ Q \cdot c^{l} = q^{l}, ~~Q \cdot c^{u} = q^{u} \\ &~&&~~c^{l}~>~ 0, ~~~~ c^{l} \in \mathbb{R}^{m}\\ &~&&~~c^{u}~>~ 0, ~~~~ c^{u} \in \mathbb{R}^{m}\\ &~&&~p_{L}~\leq~ p ~\leq~ p_{U} ~~~~ \left(p_{L}, p_{U} \in \mathbb{R}^{r+2}\right), \end{aligned}  $$

where *r* is the number of kinetic parameters, *p* is the decision vector containing the kinetic parameters *k* and the deficiency parameters *α*^*l*^ and *α*^*u*^. The lower and upper bounds for the decision variables are denoted by *p*_*L*_ and *p*_*U*_ respectively and the equilibrium manifold equations are treated as equality constraints. The values of *b*^*l*^ and *b*^*u*^, as well as the corresponding concentrations (*c*^*l*^ and *c*^*u*^) or combinations of concentrations (*q*^*l*^ and *q*^*u*^) at the bifurcation points are obtained from dose response curves. This optimization problem is non convex and multimodal and consequently a global optimization algorithm [20] is used to search for the solution.

By solving the optimization problem (13) we obtain a parameter vector *k* which is compatible with the observed dose response behavior. It is important to note here that, although we obtain a single point *k* in the parameter space as the output of the global optimization algorithm, the solution of the optimization problem (13) might not be unique, indicating that the set of parameters chosen cannot be identified univoquely. Identifiability of the parameters *a priori*, which depends on the network structure and the available observables is tackled next, where we propose a procedure to find a set of parameters and/or parameter combinations that can be identified univoquely.

### Parameter inference from dose response curves: general formulation

We have illustrated, for bistable networks of deficiency one and equilibrium manifold of dimension one, how to efficiently find a set of parameters starting from dose response curves, such that the tangent bifurcation condition is fulfilled at the two points enclosing the bistability interval in the dose response diagram. Next we generalize the method such that:

- it is valid for networks of arbitrary deficiency and manifold dimension,
- it is valid for different types of dose response curves,
- it allows to identify a set of parameters of the network with their confidence intervals starting from experimental data subject to noise.

To this aim, we first propose a numerical strategy to elucidate a priori a set of parameters or parameter combinations which can be identified univoquely from the data. Then, we use a Monte Carlo approach to estimate the parameter values and confidence intervals.

#### Error free analysis and identifiability a priori: Constraint Satisfaction Problem

In order to tackle the identifiability a priori, we consider the error free case where neither experimental nor model error is assumed, and explore the parameter space in order to find the *viable* parameter regions i.e. subsets of the parameter space where the reaction network model maintains the desired behaviour. In this case, the location of the bifurcation points of the model in the bifurcation diagram must coincide with the location observed in the experimental dose response curves. To this aim we formulate next a constraint satisfaction problem [21] where a set of constraints impose the desired conditions on the decision variables.

We define the *decision vector*$p \in \mathbb {R}^{r+2\lambda }$. It contains, on the one hand, the kinetic parameters that are unknown (*r* in case that all the rate constants are unknown) and, on the other hand, the degrees of freedom which are needed to define the equilibrium manifold at the two bifurcation points. For networks with *λ*=*δ* we take the deficiency parameter vectors $\alpha ^{l}, \alpha ^{u} \in \mathbb {R}^{\delta }$. For networks with *λ*≠*δ* a total of 2*λ* variables are taken from the vectors of deficiency parameters $\alpha ^{l}, \alpha ^{u} \in \mathbb {R}^{\delta }$ and/or the concentration vectors $c^{l}, c^{u} \in \mathbb {R}^{m}$.

A first set of (equality) constraints is given by:

(14) $$ det\left(G\left(c^{l}, \alpha^{l}; k\right)\right)=det\left(G\left(c^{u}, \alpha^{u}; k\right)\right)=0,  $$

with $k \in \mathbb {R}_{>0}^{r}$, $\alpha ^{l}, \alpha ^{u} \in \mathbb {R}^{\delta }$. A second set of (equality) constraints is given by the equilibrium manifold equations:

(15) $$ \mathcal{H}_{s}\left(c^{l}, \alpha^{l}; k\right)=0, ~~ \mathcal{H}_{s}\left(c^{u}, \alpha^{u}; k\right)=0.  $$

The mass conservation laws and the observed concentrations constitute the third set of (equality) constraints:

(16) $$ \begin{aligned} {B_{i}^{T}} \cdot c^{l} = b^{l}, &~~~~{B_{i}^{T}} \cdot c^{u} = b^{u} \\ Q \cdot c^{l} = q^{l}, &~~~~ Q \cdot c^{u} = q^{u}. \end{aligned}  $$

where the values of *b*^*l*^ and *b*^*u*^, as well as the corresponding concentrations (*c*^*l*^ and *c*^*u*^) or combinations of concentrations (*q*^*l*^ and *q*^*u*^) at the bifurcation points are obtained from dose response curves. Finally, a set of (inequality) constraints is given by

(17) $$ \begin{aligned} c^{l}~>~ 0, ~~~~& c^{l} \in \mathbb{R}^{m}\\ c^{u}~>~ 0, ~~~~& c^{u} \in \mathbb{R}^{m}\\ ~p_{L}~\leq~ p ~\leq~ p_{U} ~&~~~ \left(p_{L}, p_{U} \in \mathbb{R}^{r+2\lambda}\right), \end{aligned}  $$

where *p*_*L*_ and *p*_*U*_ are lower and upper bounds for the decision vector variables.

When the stimulus in the dose response curve is a manipulable kinetic parameter or rate constant, *k*_*i*_, two extra equality constraints need to be satisfied, indicating the values of the manipulable parameter at the lower and upper bifurcation points (${k_{i}^{l}}$ and ${k_{i}^{u}}$). The dose response curves are obtained in this case, for example, by fixing the kinetic parameter at hand at different values and measuring the corresponding steady state concentration of the species of interest.

As indicated, the dimension of the decision vector will depend on the number of kinetic parameters that remain unknown and the number of degrees of freedom that need to be fixed to define the equilibrium manifold.

In order to find the regions in the parameter space where the constraints (14-17) are satisfied we use the algorithm for exploration of parameter spaces by Zamora-Sillero *et al.* [22]. This algorithm has been shown to efficiently search for pre-defined conditions in high dimensional, non convex and poorly connected viable spaces, characteristic of complex biological networks. It combines local and global explorations of the parameter space, where for the global search, an adaptive Metropolis Monte Carlo method allows to identify poorly connected viable regions. Here, we use the algorithm to identify the *viable regions* containing those parameter vectors allowing for bistability and, in addition, compatible with the expected dose response behavior. The constraints are encoded in a cost function and the algorithm can search through the parameter space for different cost thresholds. The algorithm requires an initial viable point as an input. To start the exploration we take the optimum obtained by minimizing (in this expression *α*^1^=*α*^*l*^, *α*^2^=*α*^*u*^, *c*^1^=*c*^*l*^ and *c*^2^=*c*^*u*^):

(18) $$ J_{1}\left(k,\alpha^{1},\alpha^{2}\right) = \sum_{i=1}^{2} \left(det\left(G\left(c^{i}, \alpha^{i}; k\right)\right)\right)^{2}  $$

subject to the constraints already defined by (15-17). This optimization problem is non convex and multimodal and we solve it by means of a global optimization method [20].

The exploration algorithm provides as an output a large set of uniformly distributed viable points that allow to characterize the viable regions in the parameter space, i.e. those regions in the space of parameters that fulfill the constraints imposed [22]. The large set of viable points obtained is used to assess numerically which parameters and/or combinations of parameters are locally identifiable *a priori* starting from a given set of observables, by evaluating the viable regions obtained.

We use this approach in order to find a set of identifiable parameters in the protein activation network model. We take as observables the total enzyme concentrations at the lower and upper bifurcation points, $b^{l}={E_{T}^{l}}$ and $b^{u}={E_{T}^{u}}$ respectively, as well as the concentrations of *S*, *ESS* and *P*. The corresponding values are included in Table 1. Starting from these observables, a set of identifiable parameters *ρ*_1_,…,*ρ*_7_ (including rate constants and/or combinations of rate constants and deficiency parameters) is obtained. These identifiable parameters are shown in Table 2 together with the expressions in terms of the kinetic rate constants and the corresponding viable ranges. Here it is important to remark that, if the set of parameters is identifiable, a lower threshold in the cost function value leads to narrower viable parameter regions. As an indication of performance, for the protein activation model with its original parameterisation (ten dimensional parameter space) a single run of the exploration algorithm found 22327 viable points in 660 seconds.

**Table 1:** **Observable values for the protein activation network (error free analysis)**

${{E_{T}^{l}}}$	${{E_{T}^{u}}}$	**[** ***S*** **]** ^***l***^	**[** ***S*** **]** ^***u***^	**[** ***E*** ***S*** ***S*** **]** ^***l***^	**[** ***E*** ***S*** ***S*** **]** ^***u***^	**[** ***P*** **]** ^***l***^	**[** ***P*** **]** ^***u***^
0.007079	0.010973	1.1428	6.8362	0.0027	0.0094	8.1637	13.8571

**Table 2:** **Viable parameter regions for the protein activation network and Monte Carlo confidence intervals**

**Parameter**	**Equivalence**	**Lower bound**	**Upper bound**	**Monte Carlo confidence interval**			
*ρ*_1_	*k*_14_/*k*_41_	0.0028	0.0174	(0.0029, 0.0108)			
*ρ*_2_	*k*_18_	65.0697	530.5599	(131.4882, 275.2975)			
*ρ*_3_	*k*_26_/*k*_62_	0.4873	1.2835	(0.5230, 1.4477)			
*ρ*_4_	*k*_37_/*k*_73_	0.0652	0.0699	(0.0644, 0.06919)			
*ρ*_5_	*k*_57_	0.0160	0.0175	(0.0161, 0.0179)			
*ρ*_6_	*α*_*l*_	-0.2403	-0.2224	(-0.2446, -0.2263)			
*ρ*_7_	*α*_*u*_	-0.1424	-0.1297	(-0.1469, -0.1303)			

*k*_14_ and *k*_37_ were fixed at 1 and 0.01 respectively.

**Figure 5:**
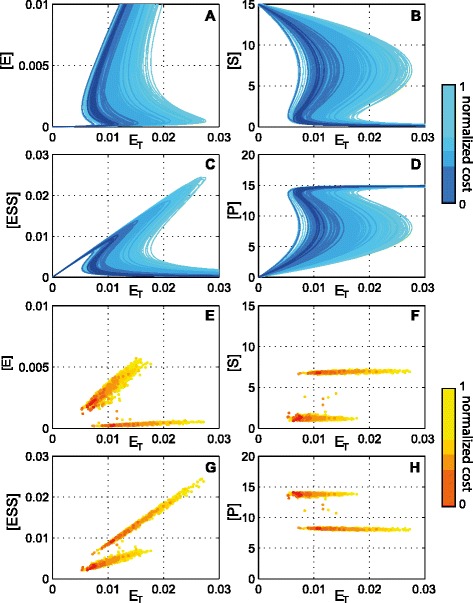
**Bifurcation diagrams and tangent bifurcation points for the protein activation mechanism.** **A-D)** Bifurcation diagrams. **E-H)** Tangent bifurcation points.

The bifurcation diagrams and tangent bifurcation points computed for the viable parameter regions are depicted in Figure 5, where the different colors indicate different thresholds of the cost value.

#### Experimental uncertainty and practical identifiability: Monte Carlo Approach

In a real experimental scenario we expect to get a number of replicate measurements for each data point which are subject to experimental error, and we need to estimate the unobserved kinetic parameters *k* from the data^e^.

Let $\hat {k}$ be a vector of estimates of *k*, and $\hat {\alpha }^{l}$, $\hat {\alpha }^{u}$ the estimated values of the deficiency parameter vectors for the lower and upper bifurcation points. The lower and upper conservation law constants can be expressed as:

$$b^{l} = {B_{i}^{T}} c\left(\hat{\alpha}^{l}; \hat{k}\right) + \varepsilon^{bl}, ~~~~b^{u} = {B_{i}^{T}} c\left(\hat{\alpha}^{u}; \hat{k}\right) + \varepsilon^{bu}, $$

where *ε*^*bl*^ and *ε*^*bu*^ represent vectors of residuals. Similarly, the expressions for the concentration *observables* read:

$$q^{l} = {Q_{i}^{T}} c\left(\hat{\alpha}^{l}; \hat{k}\right) + \varepsilon^{ql}, ~~~~q^{u} = {Q_{i}^{T}} c\left(\hat{\alpha}^{u}; \hat{k}\right) + \varepsilon^{qu}. $$

We can express the sum of squares of the residuals between the observed responses in the data set and the model prediction as a function of the kinetic parameters and deficiency parameters *α*^*l*^ and *α*^*u*^ (in this expression *α*^1^=*α*^*l*^ and *α*^2^=*α*^*u*^):

(19) $${}  {\fontsize{8.8pt}{9.6pt}\selectfont{\begin{aligned} J_{2} \left(k, \alpha^{1}, \alpha^{2}\right) = &\sum_{i=1}^{2} \left(b^{i}-B^{T}c^{i}\left(\alpha^{i}; k\right)\right)^{T}\left(b^{i}-B^{T}c^{i}\left(\alpha^{i}; k\right)\right) + \hdots \\ &\sum_{i=1}^{2} \left(q^{i}-Qc^{i}\left(\alpha^{i}; k\right)\right)^{T}\left(q^{i}-Qc^{i}\left(\alpha^{i}; k\right)\right). \end{aligned}}}  $$

We seek for estimated values of the unknown parameters that, on the one hand minimize the sum of the residuals between observed and predicted bifurcation points, and on the other hand ensure that the tangency condition is fulfilled. Therefore, the parameter estimation is formulated as an optimization problem with two individual objectives. Both the function *J*_1_ defined in Eq. (18) and the function *J*_2_ are optimized simultaneously. Since the objectives are not conflicting we can directly minimize a weighted sum of *J*_1_ and *J*_2_, with weights *w*_1_ and *w*_2_. The problem thus reads:

(20) $${} \begin{aligned} &\text{Minimize} &{}\qquad& \sum_{i=1}^{2} w_{i}J_{i} \left(k, \alpha^{l}, \alpha^{u}\right)~~\left(k\! \in\! \mathbb{R}_{>0}^{r}, \alpha^{l}, \alpha^{u} \!\in\! \mathbb{R}\right)\\ &\text{subject to:} &{} \qquad&\mathcal{H}_{s}\left(c^{l}, \alpha^{l}; k\right)=0, ~~\mathcal{H}_{s}\left(c^{u}, \alpha^{u}; k\right)=0 \\ &~&&~~c^{l}~>~ 0, ~~~ c^{l} \in \mathbb{R}^{m}\\ &~&&~~c^{u}~>~ 0, ~~~ c^{u} \in \mathbb{R}^{m}\\ &~&&~p_{L}~\leq~ p ~\leq~ p_{U} ~~~\left(p_{L}, p_{U} \in \mathbb{R}^{r+2\lambda}\right), \end{aligned}  $$

Besides the actual values of the parameters, in presence of experimental noise our goal is to obtain information on the confidence intervals of the estimated parameters. To this aim, we propose a Monte Carlo approach consisting of the following steps:

- Assuming we have enough number of experimental replicates, a probability distribution is fitted to the experimental data. Here we assume normally distributed data, but the best choice of the probability density function is to be decided upon the retrieval of the experimental data with regards to the specificities of the system under study and the data acquisition technique. Lets remind that the input data for the inverse bifurcation method are experimental measurements of the bifurcation points in dose response curves. Depending on the experimental set up, we might choose univariate or multivariate distributions to fit our data.
- A number *M* of replicates of the observed data is sampled from the distributions previously fitted. For each sample, we solve the optimization problem (20), obtaining one optimal parameter set. In this way, starting from *M* samples we obtain a distribution for every parameter.
- Statistical properties of the resulting distributions for the estimated set of parameters are calculated. Starting from the probability distribution estimates, robust confidence intervals and parameter correlations analysis can be computed [23].

As a proof of concept, we apply the approach proposed to the protein activation example with *in silico* noisy data. We take as observables, as in the previous analysis, the total enzyme concentrations at the lower and upper bifurcation points, $b^{l}={E_{T}^{l}}$ and $b^{u}={E_{T}^{u}}$, as well as the concentrations of *S*, *ESS* and *P*. In particular, we assume that we get from experimental dose response curve analysis measurements for the following pairs: $\left ({E_{T}^{l}}, [S]^{l}\right)$, $\left ({E_{T}^{l}}, [ESS]^{l}\right)$, $\left ({E_{T}^{l}}, [P]^{l}\right)$, and $\left ({E_{T}^{u}}, [S]^{u}\right)$, $\left ({E_{T}^{u}}, [ESS]^{u}\right)$, $\left ({E_{T}^{u}}, [P]^{u}\right)$. The goal is to obtain the corresponding distributions for the identifiable parameters *ρ*_1_,…,*ρ*_7_ defined in Table 2.

**Figure 6:**
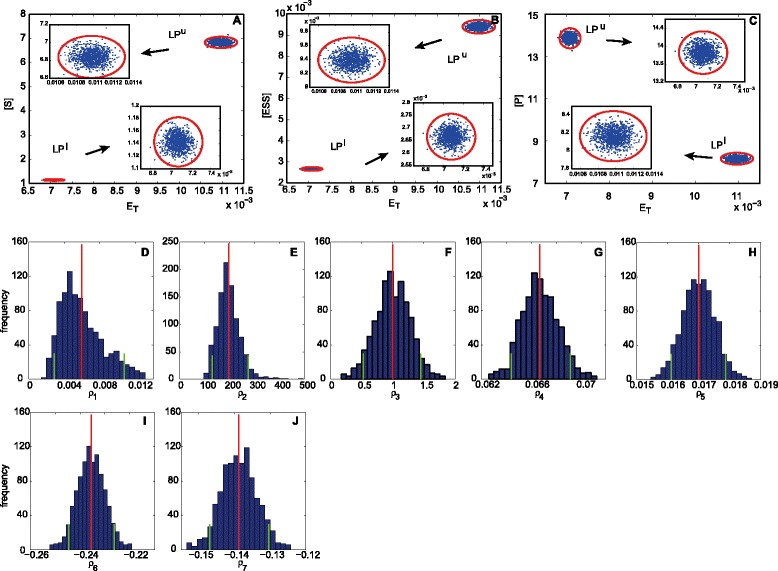
**Uncertainty propagation from the measurements to the parameters for the protein activation network.** **A-B-C)** Experimental data. The distributions obtained by fitting the original *in silico* experimental data are represented by the red ellipses (99% contour of the fitted gaussians). The blue dots are the random samples taken from the distribution. **D-E-F-G-H-I-J)** Inferred parameter distributions. Monte Carlo 95% confidence intervals are enclosed by green lines. Distribution means are indicated with red lines.

We generate the *in silico* data starting from the noise free data in Table 1, assuming normally distributed and uncorrelated error, proportional to the mean. Note that the assumptions about the error distribution and correlation might be different for different problems and experimental conditions without affecting the applicability of the method. A number of random replicates is taken from the fitted distributions in order to apply the proposed inverse bifurcation approach. The complete set of experimental data used is depicted in Figure 6A, B and C, where the fitted distributions obtained from the data are shown (we have assumed a measurement error of 10%), together with the replicate samples (1000 replicates). The optimization problem (20) is solved for each sample using the scatter search global solver by Egea *et al.* [20]. The average computation time spent by the solver in every run to get the optimal set of parameters is 30 seconds.

The histograms obtained for every parameter by applying the inverse bifurcation method proposed are shown in Figure 6D-J.

### Parameter estimation via inverse bifurcation of a genetic toggle switch

The capacity for bistability is found in some specialized gene circuits like the bacteriophage *lambda* switch [24]. The interest in genetic switches, also involving networks of non-specialized regulatory components, led to the engineering of the first toggle switch in bacteria described in [25]. In that work, the authors designed a synthetic, bistable gene circuit based on the predictions of a simple mathematical model consisting of two ordinary differential equations where the repressor binding was described by Hill functions. The conditions for bistability in the two dimensional ODE model were explored by [25,26] using nullcline analysis.

Here we are interested in the inverse problem. Starting from a gene regulatory network with experimental evidence for bistability and assuming that the underlying model structure is known, we want to estimate the kinetic parameters of the model. Through this example, we show how our inverse bifurcation method allows to exploit the capacity for bistability to infer the kinetic parameters of the network. *In silico* data are used aiming to mimic a realistic experimental scenario.

We assume that the gene regulatory network under study is shown experimentally to be bistable. In particular, a characteristic all or none hysteretic response is observed in the system when one of the decay constants is gradually varied.

**Figure 7:**
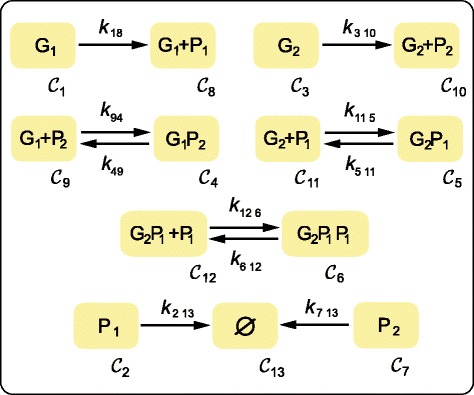
**Graph of complexes for the gene regulation toggle switch.** Every node of the graph corresponds to a network complex.

The underlying mechanism is a regulatory network with the same design principle described in [25], i.e. a repressor *P*_1_ inhibits the transcription of the repressor *P*_2_ from gene *G*_2_, and the repressor *P*_2_ inhibits the transcription of the repressor *P*_1_ from gene *G*_1_. The corresponding reaction network is depicted in Figure 7. The mutually inhibitory arrangement of the repressor genes together with the cooperativity of repression of gene *G*_2_ endow the system with capacity for bistability. Assuming mass action kinetics for all the interactions this model structure is, in qualitative terms, compatible with the experimental behavior observed.

In order to infer the parameters of the model using the inverse bifurcation method, we need to localize the tangent bifurcation points based on dose response measurements. In this case, a manipulable decay constant, the degradation rate constant corresponding to the protein *P*_2_, is chosen as the bifurcation parameter. This degradation rate constant is fixed at different values and the corresponding concentrations of different species are measured at steady state. In this case we chose as observable the concentration of proteins *P*_1_, *P*_2_ and the intermediate complex *G*_2_*P*_1_, but different selections are possible, depending on what can be measured in a particular experimental scenario.

From the dose response measurements, two tangent points are localized, at low and high decay constant values $k_{7 \,13}^{l} = 0.86$, $k_{7\, 13}^{u} = 2.96$. The steady state concentrations of the selected species (*P*_1_, *P*_2_ and *G*_2_*P*_1_) are measured at these values of the decay constant. The *in silico* experimental dose response data (99% confidence intervals of the fitted Gaussians) are shown in the figures contained in the Additional file 2, where we plot the steady state concentration measurement of each species obtained at $k_{7 \,13}^{l}$ versus the one obtained at $k_{7\, 13}^{u} $.

The gene regulation network has two mass conservation laws (all the derivations are included in Additional file 1) and the corresponding mass conservation constants are *G*_1__*T*_=*G*_2__*T*_=0.16605, computed from the gene copy numbers and the cell volume.

In order to find a set of identifiable parameters we first make use of the constraint satisfaction method proposed, starting from noise free data (we take the means of the distributions corresponding to the experimental data in Additional file 2). The numerical values for every observable are included in Table 3. The set of parameters *ρ*_1_,…,*ρ*_9_ defined in Table 4 is found to be identifiable *a priori*. For the original 14 dimensional parameter space the exploration algorithm found 23857 viable points in 990 seconds.

**Table 3:** **Observable values for gene regulation toggle switch (error free analysis)**

**[** ***P***_**1**_ **]** ^***l***^	**[** ***P***_**1**_ **]** ^***u***^	**[** ***G***_**2**_ ***P***_**1**_ **]** ^***l***^	**[** ***G***_**2**_ ***P***_**1**_ **]** ^***u***^	**[** ***P***_**2**_ **]** ^***l***^	**[** ***P***_**2**_ **]** ^***u***^
1.3714	0.1042	0.0012	0.0079	1.0180	25.5575

**Table 4:** **Optimal parameters for the gene regulation toggle switch**

**Parameter**	**Equivalence**	**Monte Carlo confidence interval**
*ρ*_1_	*k*_18_/*k*_213_	(12.7024, 22.7849)
*ρ*_2_	*k*_18_/*k*_310_	(0.0598, 0.1378)
*ρ*_3_	*k*_49_/*k*_94_	(0.6459, 1.3969)
*ρ*_4_	*k*_511_/*k*_115_	(0.5680, 1.4106)
*ρ*_5_	*k*_612_/*k*_126_	(0.0078, 0.0124)
*ρ*_6_	${\alpha _{1}^{l}}$	(63.5196, 87.2516)
*ρ*_7_	${\alpha _{2}^{l}}$	(64.0959, 87.9842)
*ρ*_8_	${\alpha _{1}^{u}}$	(0.7442,0.9986)
*ρ*_9_	${\alpha _{2}^{u}}$	(7.3285, 10.5703)

*k*_18_ was fixed at 100.

**Figure 8:**
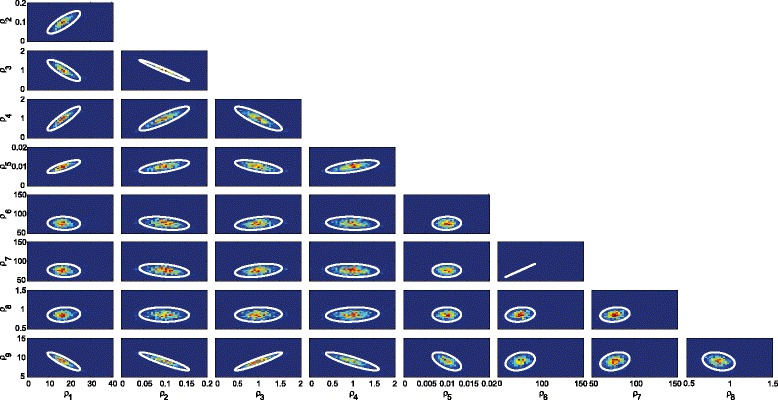
**Parameter correlations for the gene regulation toggle switch.** White contours represent the 95% pairwise confidence ellipses of the covariance matrix.

We apply the Monte Carlo approach previously introduced taking 1000 random samples from the data distributions (included in Additional file 2). The parameter distributions obtained by the inverse bifurcation method are depicted as histograms in Additional file 3. From the parameter distributions we compute the confidence intervals and the parameter correlations. The corresponding robust Monte Carlo confidence intervals are depicted together with the histograms in Additional file 3 (for the numeric values see Table 4). The two dimensional correlation among the parameters is illustrated in Figure 8. These results show the successful performance of the method for parameter estimation of the toggle switch model with *in silico* noisy data.

## Conclusions

We develop an inverse bifurcation method that allows to estimate kinetic parameters from experimental dose response curves in bistable systems. The method is suitable for a broad class of biochemical models and dose response data where the stimuli are kinetic or mass conservation constants and the responses are the steady state concentrations of species involved.

In order to infer a set of kinetic parameters of a bistable switch compatible with the available dose response experimental data, the inverse bifurcation is formulated as an optimization problem and solved by a global optimization algorithm [20].

The method exploits the inherent structural properties of biochemical reaction networks. On the one hand, inherent properties of reaction networks were used to derive (in a previous work) the ‘tangency’ condition from the reaction graph, used here instead of a generic bifurcation condition. On the other hand, the method uses the insight that tangent bifurcation points are precisely the loci where the equilibrium manifold and the reaction simplex become tangent. The tangency condition is an algebraic expression dependent on the parameters that can be systematically computed from the graph matrices. In this way our method avoids simulating the system at every iteration of the optimization algorithm reducing dramatically the computational effort in comparison to direct fitting (which in this case would entail the non trivial task of obtaining computationally the dose response curve at every iteration in order to compare it with the experimental one).

As a first approach to assess the identifiability *a priori* and select a set of parameters which are identifiable, we propose a constraint satisfaction problem. This problem is numerically solved by an algorithm [22] that allows to efficiently explore large search spaces to obtain a large set of uniformly distributed points characterizing the viable regions in the parameter space. The parameters that can be directly identified depend on the structure of the equilibrium manifold equations and the dose response data available. This first approach to identifiability is local and heuristic. A complete analytic understanding on how to find *a priori* a set of identifiable parameters is subject of future work.

In presence of experimental uncertainty, Monte Carlo simulation and global optimization are combined to generate the parameter distributions compatible with the experimentally observed behavior.

In this work we show how a set of parameters can be identified using only information about the location of the bifurcation points in the dose response curves. The inverse bifurcation method could also be applied in combination with standard approaches (for example, parameter estimation from time course data), to significantly improve identifiability and facilitate the parameter estimation task.

The method has been successfully applied to a number of examples of interest in biology including a protein activation network and a gene regulatory toggle switch, illustrating the potential of the Chemical Reaction Network Theory for kinetic model identification.

## Endnotes

^a^ In this paper we use experimental data generated *in silico*.

^b^ The time derivative $\frac {dx}{dt}(t)$ of a vector *x* depending on time *t* is denoted by $\dot {x}$. The space of *n* dimensional real vectors is denoted by $\mathbb {R}^{n}$ and the space of *m*×*n* real matrices by $\mathbb {R}^{m \times n}$. The space of *n* dimensional real vectors with all strictly positive entries is denoted by $\mathbb {R}^{n}_{>0}$ and the space of *n* dimensional real vectors with all nonnegative entries by $\mathbb {R}^{n}_{\geq 0}$.

^c^ In what follows we denote the null space (or kernel) of a matrix X by Ker(X).

^d^*D*_*x*_*F* denotes the Jacobian of *F* with respect to *x*.

^e^ We here assume for simplicity that the set of parameters *k* is identifiable. In case a different set of parameters *ρ* is found to be identifiable *a priori* the decision vector *k* is replaced by *ρ*.

## Additional files

Additional file 1
**Further details on the method for inverse bifurcation.** In this file, detailed computations for the protein activation network and the gene regulatory toggle switch examples are included.

Additional file 2
**In silico experimental data for the gene regulation toggle switch.** In this figure, the *in silico* experimental data for the gene regulation toggle switch example are depicted (red ellipses represent the 99*%* contour for the fitted gaussians) and random samples taken from the distribution (blue dots).

Additional file 3
**Parameter distribution histograms and Monte Carlo confidence intervals for the gene regulation toggle switch.** Distribution means are indicated by vertical red lines and confidence intervals (95%) are enclosed by green lines.
