# Supplementary material for: A method for inverse bifurcation of biochemical switches: inferring parameters from dose response curves
Source: BMC Syst Biol. 2014 Nov 20;8:114. doi: 10.1186/s12918-014-0114-2 (PMC4263113; doi:10.1186/s12918-014-0114-2)
Supplement: Additional file 1 — Further details on the method for inverse bifurcation. In this file, detailed computations for the protein activation network and the gene regulatory toggle switch examples are included. [file 12918_2014_114_MOESM1_ESM.pdf]

Additional File 1.
Further details on a method for inverse bifurcation of biochemical switches: inferring parameters from dose response curves

I. Otero-Muras, P. S. Yordanov, and J. Stelling

Contents

1 Protein activation module **2**
1.1 Network graph and ODE system 2
1.2 Mass conservation laws and DAE system 3
1.3 Deficiency subspace and tangent bifurcation condition 4

2 Gene regulation toggle switch **7**
2.1 Network graph and ODE system 7
2.2 Mass conservation laws and DAE system 9
2.3 Deficiency subspace and tangent bifurcation condition 10

Chapter 1

Protein activation module

1.1 Network graph and ODE system

In this section we include the derivation of the set of ODEs for the protein activation network starting from the graph of complexes. The mechanism:

$$E + S \underset{r_2}{\overset{r_1}{\rightleftharpoons}} ES \qquad\qquad ES \xrightarrow{r_3} E + P$$
$$ES + S \underset{r_5}{\overset{r_4}{\rightleftharpoons}} ESS$$

describes the activation of the protein S mediated by the protein E where S represses its own activation. Consider also the degradation of the proteins S and P, and the constitutive formation of S, described by the following reactions:

$$S \underset{r_7}{\overset{r_6}{\rightleftharpoons}} \emptyset \qquad\qquad P \xrightarrow{r_8} \emptyset$$

In total, there are five species ($m = 5$) involved in eight reactions ($r = 8$). Let us order the species such that the concentrations are $c_1 = [E]$, $c_2 = [S]$, $c_3 = [ES]$, $c_4 = [ESS]$, $c_5 = [P]$. The number of complexes is $n = 8$ and the ***graph of complexes*** is included in the main text (Fig. 1), where every complex has been labeled with a number. Note that the labeling of the complexes is arbitrary. The graph consists of $\ell = 3$ linkage classes.
In this case we chose: $\mathcal{C}_1 = ES$, $\mathcal{C}_2 = ESS$, $\mathcal{C}_3 = S$, $\mathcal{C}_4 = E + S$, $\mathcal{C}_5 = P$, $\mathcal{C}_6 = ES + S$, $\mathcal{C}_7 = \emptyset$, $\mathcal{C}_8 = E + P$.
The molecularity matrix $Y \in \mathbb{R}^{m\times n}$ as defined in the main text, contains the molecularities of the species involved in every complex:

$$Y = \begin{bmatrix} 0 & 0 & 0 & 1 & 0 & 0 & 0 & 1 \\ 0 & 0 & 1 & 1 & 0 & 1 & 0 & 0 \\ 1 & 0 & 0 & 0 & 0 & 1 & 0 & 0 \\ 0 & 1 & 0 & 0 & 0 & 0 & 0 & 0 \\ 0 & 0 & 0 & 0 & 1 & 0 & 0 & 1 \end{bmatrix}. \qquad (1.1)$$

Table 1.1: Reaction rates

reaction	rate	reaction rate
r_1	v_{41}	$k_{41}c_1c_2$
r_2	v_{14}	$k_{14}c_3$
r_3	v_{18}	$k_{18}c_3$
r_4	v_{62}	$k_{62}c_2c_3$
r_5	v_{26}	$k_{26}c_4$
r_6	v_{37}	$k_{37}c_2$
r_7	v_{73}	k_{73}
r_8	v_{57}	$k_{57}c_5$

The vector $\psi(c) \in \mathbb{R}^n_{\geq 0}$ containing the mass action monomials associated to the complexes (Eq. 1 in the main text) is:

$$\psi(c) = (c_3,\ c_4,\ c_2,\ c_1c_2,\ c_5,\ c_2c_3,\ 1,\ c_1c_5)^T, \tag{1.2}$$

and the reaction rates of the network are shown in Table 1.1.
The matrix $A \in \mathbb{R}^{n\times n}$ is:

$$A = \begin{bmatrix} \text{-}(k_{14}+k_{18}) & 0 & 0 & k_{41} & 0 & 0 & 0 & 0 \\ 0 & \text{-}k_{26} & 0 & 0 & 0 & k_{62} & 0 & 0 \\ 0 & 0 & \text{-}k_{37} & 0 & 0 & 0 & k_{73} & 0 \\ k_{14} & 0 & 0 & \text{-}k_{41} & 0 & 0 & 0 & 0 \\ 0 & 0 & 0 & 0 & \text{-}k_{57} & 0 & 0 & 0 \\ 0 & k_{26} & 0 & 0 & 0 & \text{-}k_{62} & 0 & 0 \\ 0 & 0 & k_{37} & 0 & k_{57} & 0 & \text{-}k_{73} & 0 \\ k_{18} & 0 & 0 & 0 & 0 & 0 & 0 & 0 \end{bmatrix}$$

and the set of ODEs describing the dynamics (corresponding to Eq. 2 in the main text) reads:

$$\begin{aligned} \dot{c}_1 &= (k_{14} + k_{18})c_3 - k_{41}c_1c_2 \\ \dot{c}_2 &= k_{14}c_3 + k_{26}c_4 - k_{37}c_2 - k_{41}c_1c_2 - k_{62}c_2c_3 + k_{73} \\ \dot{c}_3 &= (-k_{14} - k_{18})c_3 + k_{26}c_4 + k_{41}c_1c_2 - k_{62}c_2c_3 \\ \dot{c}_4 &= -k_{26}c_4 + k_{62}c_2c_3 \\ \dot{c}_5 &= k_{18}c_3 - k_{57}c_5. \end{aligned} \tag{1.3}$$

1.2 Mass conservation laws and DAE system

In this section we include the derivation of the DAE system for the protein activation network. The stoichiometric subspace is spanned by the following vectors:

$$\mathcal{S} = span\left\{ \begin{pmatrix} 1 \\ 1 \\ -1 \\ 0 \\ 0 \end{pmatrix}, \begin{pmatrix} 1 \\ 0 \\ -1 \\ 0 \\ 1 \end{pmatrix}, \begin{pmatrix} 0 \\ -1 \\ -1 \\ 1 \\ 0 \end{pmatrix}, \begin{pmatrix} 0 \\ 0 \\ 0 \\ 0 \\ 1 \end{pmatrix} \right\}, \tag{1.4}$$

the dimension of the stoichiometric subspace is $s = 4$ and thus we have one conservation relation $(m - s = 1)$.

The left null space of YA is:

$$B^T = \left(\begin{array}{ccccc} 1 & 0 & 1 & 1 & 0 \end{array} \right). \tag{1.5}$$

The function W (defined by Eq. 3 in the main text) reads:

$$W(c; c_0) = c_1 + c_3 + c_4 - c_{1_0} - c_{3_0} - c_{4_0}, \tag{1.6}$$

and the reaction polyhedron consists of all $c \geq 0$ such that $W(c; c_0) = 0$. In other words, there is one conservation law in the system (Eq. 4 in the main text) given by:

$$c_1 + c_3 + c_4 = c_{1_0} - c_{3_0} - c_{4_0}. \tag{1.7}$$

Note that the right hand side of this expression is the total amount of enzyme E_T:

$$E_T = c_{1_0} - c_{3_0} - c_{4_0}. \tag{1.8}$$

The existence of conservation laws implies that the ODE system (1.3) is not minimal. We can partition the state space to get a minimal set of ODEs and a set of algebraic equations, obtaining the following DAE system consisting of 4 linearly independent ODEs and one algebraic equation:

$$\begin{aligned}
c_3 &= E_T - c_1 - c_4 \\
\dot{c}_1 &= (k_{14} + k_{18})c_3 - k_{41}c_1c_2 \\
\dot{c}_2 &= k_{14}c_3 + k_{26}c_4 - k_{37}c_2 - k_{41}c_1c_2 - k_{62}c_2c_3 + k_{73} \\
\dot{c}_4 &= -k_{26}c_4 + k_{62}c_2c_3, \\
\dot{c}_5 &= k_{18}c_3 - k_{57}c_5
\end{aligned}$$

1.3 Deficiency subspace and tangent bifurcation condition

In this section we compute the deficiency subspace, the equilibrium manifold equations and the tangent bifurcation condition for the protein activation network. The deficiency of the network is one

$$\delta = 8 - 3 - 4 = 1$$

and, therefore, the deficiency subspace is one dimensional. In order to compute a basis for the deficiency subspace let us first define a $n \times \ell$ matrix Λ where the entry (i, j) corresponds to the node $\mathcal{C}_i$ in the linkage class $\mathcal{L}_j$, being:

$\Lambda_{i,j} = 1$ if $\mathcal{C}_i \in \mathcal{L}_j$

$\Lambda_{i,j} = 0$ otherwise.

as indicated in the main text. The matrix Λ results:

$$\Lambda = \begin{bmatrix} 1 & 0 & 0 \\ 0 & 1 & 0 \\ 0 & 0 & 1 \\ 1 & 0 & 0 \\ 0 & 0 & 1 \\ 0 & 1 & 0 \\ 0 & 0 & 1 \\ 1 & 0 & 0 \end{bmatrix}. \tag{1.9}$$

A basis ω is computed taking into account Eq. 6 in the main text, obtaining:

$$\omega = \begin{pmatrix} 0 & 0 & -1 & 1 & 1 & 0 & 0 & -1 \end{pmatrix}^T. \tag{1.10}$$

At equilibrium the following equality (Eq. 7 in the main text, with $\delta = 1$) must hold:

$$A\psi(c) = \alpha\omega \tag{1.11}$$

obtaining:

$$\begin{aligned}
\psi_4(c) &= k_{14}/k_{41}\psi_1(c) - \alpha/k_{41} \\
\psi_5(c) &= -\alpha/k_{57} \\
\psi_6(c) &= k_{26}/k_{62}\psi_2(c) \\
\psi_7(c) &= k_{37}/k_{73}\psi_3(c) - \alpha/k_{73} \\
\psi_1(c) &= -\alpha/k_{18}
\end{aligned}$$

Using (1.2) we can write the previous set of equations in terms of the concentrations:

$$\begin{aligned}
c_1c_2 &= k_{14}/k_{41}c_3 - \alpha/k_{41} \\
c_5 &= -\alpha/k_{57} \\
c_2c_3 &= k_{26}/k_{62}c_4 \\
1 &= k_{37}/k_{73}c_2 - \alpha/k_{73} \\
c_3 &= -\alpha/k_{18}.
\end{aligned} \tag{1.12}$$

The locus of equilibria in the space of the species (Eq. 9 in the main text) is given by:

$$\begin{aligned}
\mathcal{H}_{s_1} &= k_{14}/k_{41}c_3 - \alpha/k_{41} - c_1c_2, \\
\mathcal{H}_{s_2} &= c_5 + \alpha/k_{57}, \\
\mathcal{H}_{s_3} &= k_{26}/k_{62}c_4 - c_2c_3, \\
\mathcal{H}_{s_4} &= k_{37}/k_{73}c_2 - \alpha/k_{73} - 1, \\
\mathcal{H}_{s_5} &= c_3 + \alpha/k_{18}.
\end{aligned}$$

Let us compute the Jacobian of $\mathcal{H}_s$ with respect to c:

$$D_c\mathcal{H}_s = \begin{pmatrix} -c_2 & -c_1 & k_{14}/k_{41} & 0 & 0 \\ 0 & 0 & 0 & 0 & 1 \\ 0 & -c_3 & -c_2 & k_{26}/k_{62} & 0 \\ 0 & k_{37}/k_{73} & 0 & 0 & 0 \\ 0 & 0 & 1 & 0 & 0 \end{pmatrix}, \tag{1.13}$$

and the Jacobian of $\mathcal{H}_s$ with respect to α is:

$$D_\alpha\mathcal{H}_s = \begin{pmatrix} -1/k_{41} \\ 1/k_{57} \\ 0 \\ -1/k_{73} \\ 1/k_{18} \end{pmatrix}. \tag{1.14}$$

The Jacobian of W with respect to c is:

$$D_cW = \begin{pmatrix} 1 & 0 & 1 & 1 & 0 \end{pmatrix}, \tag{1.15}$$

and the Jacobian of W with respect to α is 0. From Eq. 10 in the main text, we get:

$$\frac{dc}{d\alpha}(\alpha, k) = \begin{pmatrix} -1/(k_{14}c_2) - c_1/(k_{37}c_2) - k_{14}/(k_{41}k_{18}c_2) \\ 1/k_{37} \\ -1/k_{18} \\ k_{62}c_3/(k_{37}k_{26}) - c_2k_{62}/(k_{26}k_{18}) \\ -1/k_{57} \end{pmatrix}, \tag{1.16}$$

and the matrix G, given by Eq. 11 from the main text reads:

$$G(c, \alpha, k) = \begin{pmatrix} -c_2 & -c_1 & k_{14}/k_{41} & 0 & 0 & -1/k_{41} \\ 0 & 0 & 0 & 0 & 1 & 1/k_{57} \\ 0 & -c_3 & -c_2 & k_{26}/k_{62} & 0 & 0 \\ 0 & k_{37}/k_{73} & 0 & 0 & 0 & -1/k_{73} \\ 0 & 0 & 1 & 0 & 0 & 1/k_{18} \\ 1 & 0 & 1 & 1 & 0 & 0 \end{pmatrix}.$$

Computing the determinant of the matrix G, Eq. 12 from the main text (condition for multistationarity) reads:

$$c_2c_3 - \frac{k_{26}k_{37}}{k_{62}k_{41}} - \frac{k_{26}k_{37}k_{14}}{k_{62}k_{41}k_{18}} - \frac{k_{26}k_{37}c_2}{k_{62}k_{41}} - \frac{k_{26}c_1}{k_{62}} - \frac{k_{37}c_2^2}{k_{18}} = 0.$$

Chapter 2

Gene regulation toggle switch

2.1 Network graph and ODE system

In this section we include the derivation of the set of ODEs for the gene regulation switch example starting from the graph of complexes. The gene regulatory network under study consists of the following set of reactions:

$$G_1 \xrightarrow{r_1} G_1 + P_1 \qquad\qquad G_2 \xrightarrow{r_2} G_2 + P_2$$

$$G_1 + P_2 \underset{r_4}{\overset{r_3}{\rightleftharpoons}} G_1P_2 \qquad\qquad G_2 + P_1 \underset{r_6}{\overset{r_5}{\rightleftharpoons}} G_2P_1$$

$$G_2P_1 + P_1 \underset{r_8}{\overset{r_7}{\rightleftharpoons}} G_2P_1P_1$$

$$P_1 \xrightarrow{r_9} \emptyset \xleftarrow{r_{10}} P_2$$

In total, there are seven species ($m = 7$) involved in ten reactions ($r = 10$). Let us order the species such that the concentrations are $c_1 = [G_1]$, $c_2 = [P_1]$, $c_3 = [G_2]$, $c_4 = [G_1P_2]$, $c_5 = [G_2P_1]$, $c_6 = [G_2P_1P_1]$, $c_7 = [P_2]$. The number of complexes is $n = 13$, and the *graph of complexes* is depicted in Fig. 7 from the main text, where every complex has been labeled with a number. Note that the labeling of the complexes is arbitrary. The graph consists of $\ell = 6$ linkage classes. In this case we select the labels indicated in Table 2.1.
The molecularity matrix $Y \in \mathbb{R}^{7\times 13}$ contains the molecularities of the species involved in every complex:

$$Y = \begin{bmatrix} 1 & 0 & 0 & 0 & 0 & 0 & 0 & 1 & 1 & 0 & 0 & 0 & 0 \\ 0 & 1 & 0 & 0 & 0 & 0 & 0 & 1 & 0 & 0 & 1 & 1 & 0 \\ 0 & 0 & 1 & 0 & 0 & 0 & 0 & 0 & 0 & 1 & 1 & 0 & 0 \\ 0 & 0 & 0 & 1 & 0 & 0 & 0 & 0 & 0 & 0 & 0 & 0 & 0 \\ 0 & 0 & 0 & 0 & 1 & 0 & 0 & 0 & 0 & 0 & 0 & 1 & 0 \\ 0 & 0 & 0 & 0 & 0 & 1 & 0 & 0 & 0 & 0 & 0 & 0 & 0 \\ 0 & 0 & 0 & 0 & 0 & 0 & 1 & 0 & 1 & 1 & 0 & 0 & 0 \end{bmatrix}. \tag{2.1}$$

The vector $\psi(c) \in \mathbb{R}^n_{\geq 0}$, as defined by Eq. 1 in the main text, contains the mass

Table 2.1: Complexes labeling

$\mathcal{C}_1$	G_1
$\mathcal{C}_2$	P_1
$\mathcal{C}_3$	G_2
$\mathcal{C}_4$	G_1P_2
$\mathcal{C}_5$	G_2P_1
$\mathcal{C}_6$	$G_2P_1P_1$
$\mathcal{C}_7$	P_2
$\mathcal{C}_8$	G_1+P_1
$\mathcal{C}_9$	G_1+P_2
$\mathcal{C}_{10}$	G_2+P_2
$\mathcal{C}_{11}$	G_2+P_1
$\mathcal{C}_{12}$	$G_2P_1+P_1$
$\mathcal{C}_{13}$	$\emptyset$

Table 2.2: Reaction rates

reaction	rate	reaction rate
r_1	$v_{1\,8}$	$k_{1\,8}c_1$
r_2	$v_{3\,10}$	$k_{3\,10}c_3$
r_3	$v_{9\,4}$	$k_{9\,4}c_1c_7$
r_4	$v_{4\,9}$	$k_{4\,9}c_4$
r_5	$v_{11\,5}$	$k_{11\,5}c_3c_2$
r_6	$v_{5\,11}$	$k_{5\,11}c_5$
r_7	$v_{12\,6}$	$k_{12\,6}c_5c_2$
r_8	$v_{6\,12}$	$k_{6\,12}c_6$
r_9	$v_{2\,13}$	$k_{2\,13}c_2$
r_{10}	$v_{7\,13}$	$k_{7\,13}c_7$

action monomials associated to the complexes:

$$\psi(c) = (c_1,\ c_2,\ c_3,\ c_4,\ c_5,\ c_6,\ c_7,\ c_1c_2,\ c_1c_7,\ c_3c_7,\ c_2c_3,\ c_2c_5,\ 1)^T, \tag{2.2}$$

and the reaction rates of the network are shown in Table 2.2.
The matrix $A \in \mathbb{R}^{n\times n}$ is:

$$A = \begin{bmatrix}
-k_{1\,8} & 0 & 0 & 0 & 0 & 0 & 0 & 0 & 0 & 0 & 0 & 0 & 0\\
0 & -k_{2\,13} & 0 & 0 & 0 & 0 & 0 & 0 & 0 & 0 & 0 & 0 & 0\\
0 & 0 & -k_{3\,10} & 0 & 0 & 0 & 0 & 0 & 0 & 0 & 0 & 0 & 0\\
0 & 0 & 0 & -k_{4\,9} & 0 & 0 & 0 & 0 & k_{9\,4} & 0 & 0 & 0 & 0\\
0 & 0 & 0 & 0 & -k_{5\,11} & 0 & 0 & 0 & 0 & 0 & k_{11\,5} & 0 & 0\\
0 & 0 & 0 & 0 & 0 & -k_{6\,12} & 0 & 0 & 0 & 0 & 0 & k_{12\,6} & 0\\
0 & 0 & 0 & 0 & 0 & 0 & -k_{7\,13} & 0 & 0 & 0 & 0 & 0 & 0\\
k_{1\,8} & 0 & 0 & 0 & 0 & 0 & 0 & 0 & 0 & 0 & 0 & 0 & 0\\
0 & 0 & 0 & k_{4\,9} & 0 & 0 & 0 & 0 & -k_{9\,4} & 0 & 0 & 0 & 0\\
0 & 0 & k_{3\,10} & 0 & 0 & 0 & 0 & 0 & 0 & 0 & 0 & 0 & 0\\
0 & 0 & 0 & 0 & k_{5\,11} & 0 & 0 & 0 & 0 & 0 & -k_{11\,5} & 0 & 0\\
0 & 0 & 0 & 0 & 0 & k_{6\,12} & 0 & 0 & 0 & 0 & 0 & -k_{12\,6} & 0\\
0 & k_{2\,13} & 0 & 0 & 0 & 0 & k_{7\,13} & 0 & 0 & 0 & 0 & 0 & 0
\end{bmatrix},$$

and the set of ODEs corresponding to Eq. 2 in the main text reads:

$$\begin{aligned}
\dot{c}_1 &= k_{4\,9}c_4 - k_{9\,4}c_1c_7 \\
\dot{c}_2 &= k_{1\,8}c_1 - k_{2\,13}c_2 + k_{5\,11}c_5 + k_{6\,12}c_6 - k_{11\,5}c_2c_3 - k_{12\,6}c_2c_5 \\
\dot{c}_3 &= k_{5\,11}c_5 - k_{11\,5}c_2c_3 \\
\dot{c}_4 &= -k_{4\,9}c_4 + k_{9\,4}c_1c_7 \\
\dot{c}_5 &= -k_{5\,11}c_5 + k_{6\,12}c_6 + k_{11\,5}c_2c_3 - k_{12\,6}c_2c_5 \\
\dot{c}_6 &= -k_{6\,12}c_6 + k_{12\,6}c_2c_5 \\
\dot{c}_7 &= k_{3\,10}c_3 + k_{4\,9}c_4 - k_{7\,13}c_7 - k_{9\,4}c_1c_7.
\end{aligned} \tag{2.3}$$

2.2 Mass conservation laws and DAE system

In this section we include the derivation the DAE system for the gene regulation switch example starting from the graph of complexes. The stoichiometric subspace is spanned by the following vectors:

$$\mathcal{S} = span \left\{ \begin{pmatrix} 0 \\ 1 \\ 0 \\ 0 \\ 0 \\ 0 \\ 0 \end{pmatrix}, \begin{pmatrix} 0 \\ 0 \\ 0 \\ 0 \\ 0 \\ 0 \\ 1 \end{pmatrix}, \begin{pmatrix} 1 \\ 0 \\ 0 \\ -1 \\ 0 \\ 0 \\ 1 \end{pmatrix}, \begin{pmatrix} 0 \\ 1 \\ 1 \\ 0 \\ -1 \\ 0 \\ 0 \end{pmatrix}, \begin{pmatrix} 0 \\ 1 \\ 0 \\ 0 \\ 1 \\ -1 \\ 0 \end{pmatrix} \right\}. \tag{2.4}$$

The dimension of the stoichiometric subspace is $s = 5$ and thus we have two conservation relation ($m - s = 2$).
The left null space of YA is:

$$B^T = \begin{pmatrix} 1 & 0 & 0 & 1 & 0 & 0 & 0 \\ 0 & 0 & 1 & 0 & 1 & 1 & 0 \end{pmatrix}. \tag{2.5}$$

The function defined by Eq. 3 in the main text reads:

$$\begin{aligned}
W_1(c; c_0) &= c_1 + c_4 - c_{1_0} - c_{4_0} \\
W_2(c; c_0) &= c_3 + c_5 + c_6 - c_{3_0} - c_{5_0} - c_{6_0}
\end{aligned}$$

and the reaction polyhedron consists of all $c \geq 0$ such that $W(c; c_0) = 0$. In other words, there are two conservation laws in the system (Eq. 4 in the main text) given by:

$$\begin{aligned}
c_1 + c_4 &= c_{1_0} + c_{4_0}, \\
c_3 + c_5 + c_6 &= c_{3_0} + c_{5_0} + c_{6_0}.
\end{aligned} \tag{2.6}$$

Note that on the right hand side of these equations we have the total amount of G_1 and G_2, respectively:

$$\begin{aligned}
G_{1_T} &= c_{1_0} + c_{4_0}, \\
G_{2_T} &= c_{3_0} + c_{5_0} + c_{6_0}.
\end{aligned}$$

The existence of conservation laws implies that the ODE system given by (2.3) is not minimal. We can partition the state space to get a minimal set of 5 linearly independent ODEs and a set of 2 algebraic equations obtaining the following DAE system:

$$\begin{aligned}
c_4 &= G_{1_T} - c_1 \\
c_6 &= G_{2_T} - c_3 - c_5 \\
\dot{c}_1 &= k_{4\,9}c_4 - k_{9\,4}c_1c_7 \\
\dot{c}_2 &= k_{1\,8}c_1 - k_{2\,13}c_2 + k_{5\,11}c_5 + k_{6\,12}c_6 - k_{11\,5}c_2c_3 - k_{12\,6}c_2c_5 \\
\dot{c}_3 &= k_{5\,11}c_5 - k_{11\,5}c_2c_3 \\
\dot{c}_5 &= -k_{5\,11}c_5 + k_{6\,12}c_6 + k_{11\,5}c_2c_3 - k_{12\,6}c_2c_5 \\
\dot{c}_7 &= k_{3\,10}c_3 + k_{4\,9}c_4 - k_{7\,13}c_7 - k_{9\,4}c_1c_7.
\end{aligned}$$

2.3 Deficiency subspace and tangent bifurcation condition

In this section we compute the deficiency subspace, the equilibrium manifold equations and the tangent bifurcation condition for the gene regulatory network. The deficiency of the network is two

$$\delta = 13 - 6 - 5 = 2$$

and, therefore, the deficiency subspace is two dimensional. In order to compute a basis for the deficiency subspace let us first build the $n \times \ell$ matrix Λ where the entry (i, j) corresponds to the node $\mathcal{C}_i$ in the linkage class $\mathcal{L}_j$, being

$$\Lambda_{i,j} = 1 \text{ if } \mathcal{C}_i \in \mathcal{L}_j$$

$$\Lambda_{i,j} = 0 \text{ otherwise.}$$

as indicated in the main text. The matrix Λ results:

$$\Lambda = \begin{bmatrix} 1 & 0 & 0 & 0 & 0 & 0 \\ 0 & 0 & 0 & 0 & 0 & 1 \\ 0 & 1 & 0 & 0 & 0 & 0 \\ 0 & 0 & 1 & 0 & 0 & 0 \\ 0 & 0 & 0 & 1 & 0 & 0 \\ 0 & 0 & 0 & 0 & 1 & 0 \\ 0 & 0 & 0 & 0 & 0 & 1 \\ 1 & 0 & 0 & 0 & 0 & 0 \\ 0 & 0 & 1 & 0 & 0 & 0 \\ 0 & 1 & 0 & 0 & 0 & 0 \\ 0 & 0 & 0 & 1 & 0 & 0 \\ 0 & 0 & 0 & 0 & 1 & 0 \\ 0 & 0 & 0 & 0 & 0 & 1 \end{bmatrix}. \tag{2.7}$$

A basis ω is computed taking into account Eq. 6 in the main text, obtaining:

$$\omega_1 = \begin{pmatrix} 1 & 1 & -1 & 0 & 0 & 0 & -1 & -1 & 0 & 1 & 0 & 0 & 0 \end{pmatrix}^T \tag{2.8}$$

$$\omega_2 = \begin{pmatrix} -1 & -1 & 0 & 0 & 0 & 0 & 0 & 1 & 0 & 0 & 0 & 0 & 1 \end{pmatrix}^T. \tag{2.9}$$

At equilibrium, Eq. 7 in the main text (with $\delta = 2$) must hold:

$$A\psi(c) = \alpha_1\omega_1 + \alpha_2\omega_2 \tag{2.10}$$

obtaining:

$$\begin{aligned}
\psi_1(c) &= (\alpha_2 - \alpha_1)/k_{1\,8} \\
\psi_2(c) &= (\alpha_2 - \alpha_1)/k_{2\,13} \\
\psi_3(c) &= \alpha_1/k_{3\,10} \\
\psi_4(c) &= k_{9\,4}/k_{4\,9}\psi_9(c) \\
\psi_5(c) &= k_{11\,5}/k_{5\,11}\psi_{11}(c) \\
\psi_6(c) &= k_{12\,6}/k_{6\,12}\psi_{12}(c) \\
\psi_7(c) &= \alpha_1/k_{7\,13}.
\end{aligned}$$

Using (2.2) the previous equations can be written in terms of the concentrations:

$$\begin{aligned}
c_1 &= (\alpha_2 - \alpha_1)/k_{1\,8} \\
c_2 &= (\alpha_2 - \alpha_1)/k_{2\,13} \\
c_3 &= \alpha_1/k_{3\,10} \\
c_4 &= k_{9\,4}/k_{4\,9}c_1c_7 \\
c_5 &= k_{11\,5}/k_{5\,11}c_2c_3 \\
c_6 &= k_{12\,6}/k_{6\,12}c_2c_5 \\
c_7 &= \alpha_1/k_{7\,13}.
\end{aligned} \tag{2.11}$$

The locus of equilibria in the space of the species (Eq. 9 in the main text) is given by:

$$\begin{aligned}
\mathcal{H}_{s_1} &= c_1 - (\alpha_2 - \alpha_1)/k_{1\,8} \\
\mathcal{H}_{s_2} &= c_2 - (\alpha_2 - \alpha_1)/k_{2\,13} \\
\mathcal{H}_{s_3} &= c_3 - \alpha_1/k_{3\,10} \\
\mathcal{H}_{s_4} &= c_1c_7 - k_{4\,9}/k_{9\,4}c_4 \\
\mathcal{H}_{s_5} &= c_2c_3 - k_{5\,11}/k_{11\,5}c_5 \\
\mathcal{H}_{s_6} &= c_2c_5 - k_{6\,12}/k_{12\,6}c_6 \\
\mathcal{H}_{s_7} &= c_7 - \alpha_1/k_{7\,13}.
\end{aligned}$$

Let us compute the Jacobian of $\mathcal{H}_s$ with respect to c:

$$D_c\mathcal{H}_s = \begin{pmatrix}
1 & 0 & 0 & 0 & 0 & 0 & 0 \\
0 & 1 & 0 & 0 & 0 & 0 & 0 \\
0 & 0 & 1 & 0 & 0 & 0 & 0 \\
c_7 & 0 & 0 & -k_{4\,9}/k_{9\,4} & 0 & 0 & c_1 \\
0 & c_3 & c_2 & 0 & -k_{5\,11}/k_{11\,5} & 0 & 0 \\
0 & c_5 & 0 & 0 & c_2 & -k_{6\,12}/k_{12\,6} & 0 \\
0 & 0 & 0 & 0 & 0 & 0 & 1
\end{pmatrix}, \tag{2.12}$$

and the Jacobian of $\mathcal{H}_s$ with respect to α is:

$$D_\alpha \mathcal{H}_s = \begin{pmatrix} 1/k_{1\,8} & -1/k_{1\,8} \\ 1/k_{2\,13} & -1/k_{2\,13} \\ -1/k_{3\,10} & 0 \\ 0 & 0 \\ 0 & 0 \\ 0 & 0 \\ -1/k_{7\,13} & 0 \end{pmatrix}. \tag{2.13}$$

The Jacobian of W with respect to c is:

$$D_c W = \begin{pmatrix} 1 & 0 & 0 & 1 & 0 & 0 & 0 \\ 0 & 0 & 1 & 0 & 1 & 1 & 0 \end{pmatrix}, \tag{2.14}$$

and the Jacobian of W with respect to α is the zero vector $D_\alpha W = (0, 0)$. Therefore, the matrix G given by Eq. 11 in the main text reads:

$$G(c, \alpha, k) = \begin{pmatrix} 1 & 0 & 0 & 0 & 0 & 0 & 0 & \frac{1}{k_{1\,8}} & -\frac{1}{k_{1\,8}} \\ 0 & 1 & 0 & 0 & 0 & 0 & 0 & \frac{1}{k_{2\,13}} & -\frac{1}{k_{2\,13}} \\ 0 & 0 & 1 & 0 & 0 & 0 & 0 & -\frac{1}{k_{3\,10}} & 0 \\ c_7 & 0 & 0 & -\frac{k_{4\,9}}{k_{9\,4}} & 0 & 0 & c_1 & 0 & 0 \\ 0 & c_3 & c_2 & 0 & -\frac{k_{5\,11}}{k_{11\,5}} & 0 & 0 & 0 & 0 \\ 0 & c_5 & 0 & 0 & c_2 & -\frac{k_{6\,12}}{k_{12\,6}} & 0 & 0 & 0 \\ 0 & 0 & 0 & 0 & 0 & 0 & 1 & -\frac{1}{k_{7\,13}} & 0 \\ 1 & 0 & 0 & 1 & 0 & 0 & 0 & 0 & 0 \\ 0 & 0 & 1 & 0 & 1 & 1 & 0 & 0 & 0 \end{pmatrix}. \tag{2.15}$$

Computing the determinant of the matrix G and applying Eq. 12 in the main text, we obtain the condition for multistationarity.

Bibliography

[1] Otero-Muras I, Banga JR, Alonso AA (2012). Characterizing Multistationarity Regimes in Biochemical Reaction Networks. PLoS ONE 7(7): e39194.

[2] Egea JA, Rodriguez-Fernandez M, Banga JR and Marti R(2007). Scatter Search for chemical and bioprocess optimization. *J. Global Optim.*, 37(3):481–503.
