# Supplementary material for: A method for inverse bifurcation of biochemical switches: inferring parameters from dose response curves
Source: BMC Syst Biol. 2014 Nov 20;8:114. doi: 10.1186/s12918-014-0114-2 (PMC4263113; doi:10.1186/s12918-014-0114-2)
Supplement: Additional file 2 — In silico experimental data for the gene regulation toggle switch. In this figure, the in silico experimental data for the gene regulation toggle switch example are depicted (red ellipses represent the 99% contour for the fitted gaussians) and random samples taken from the distribution (blue dots). [file 12918_2014_114_MOESM2_ESM.pdf]

Additional File 2

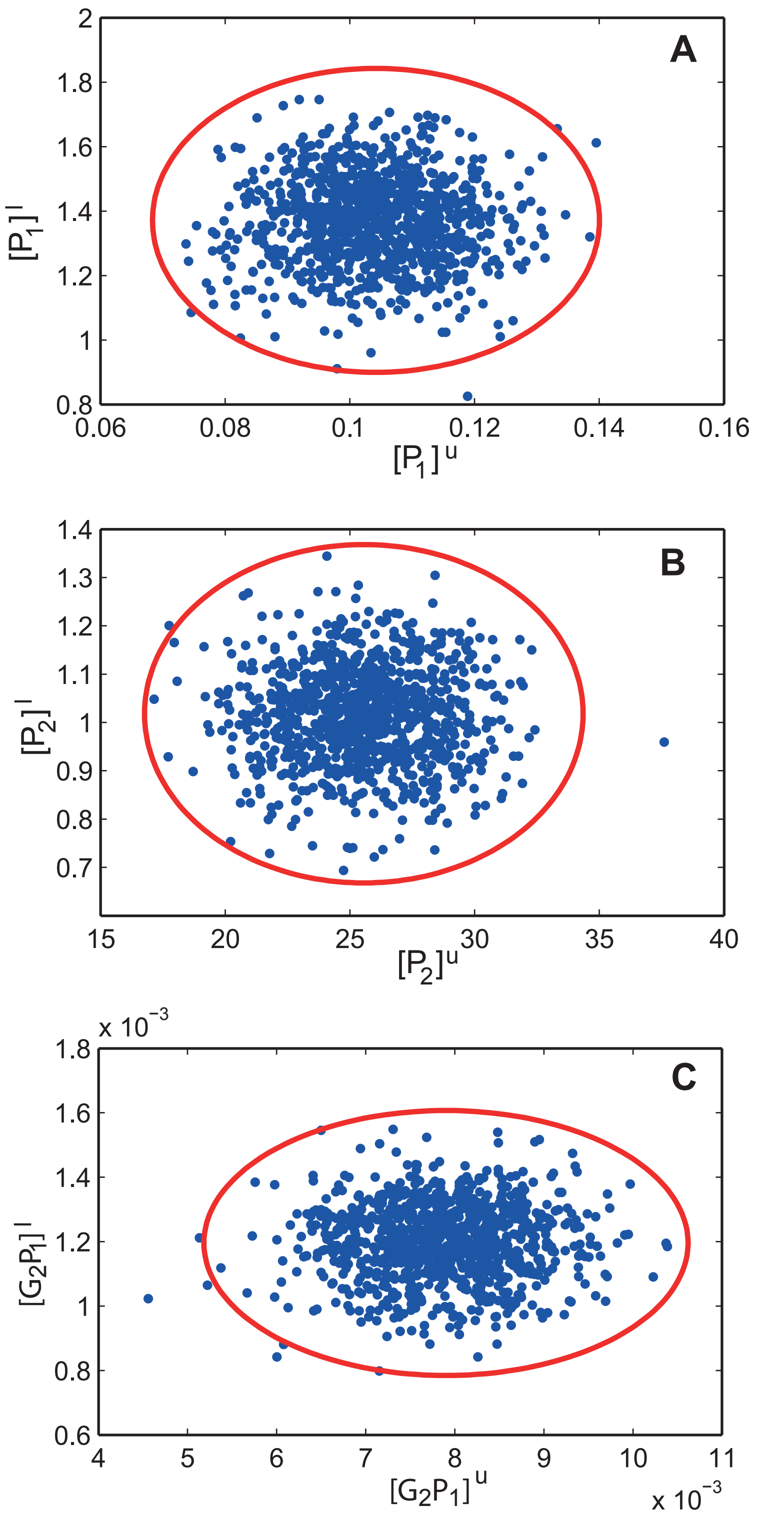

In silico experimental for the data gene regulation toggle switch example (red ellipses represent the 99% contour for the fitted gaussians) and random samples taken from the distribution (blue dots).
