# Supplementary material for: A method for inverse bifurcation of biochemical switches: inferring parameters from dose response curves
Source: BMC Syst Biol. 2014 Nov 20;8:114. doi: 10.1186/s12918-014-0114-2 (PMC4263113; doi:10.1186/s12918-014-0114-2)
Supplement: Additional file 3 — Parameter distribution histograms and Monte Carlo confidence intervals for the gene regulation toggle switch. Distribution means are indicated by vertical red lines and confidence intervals (95%) are enclosed by green lines. [file 12918_2014_114_MOESM3_ESM.pdf]

Additional File 3

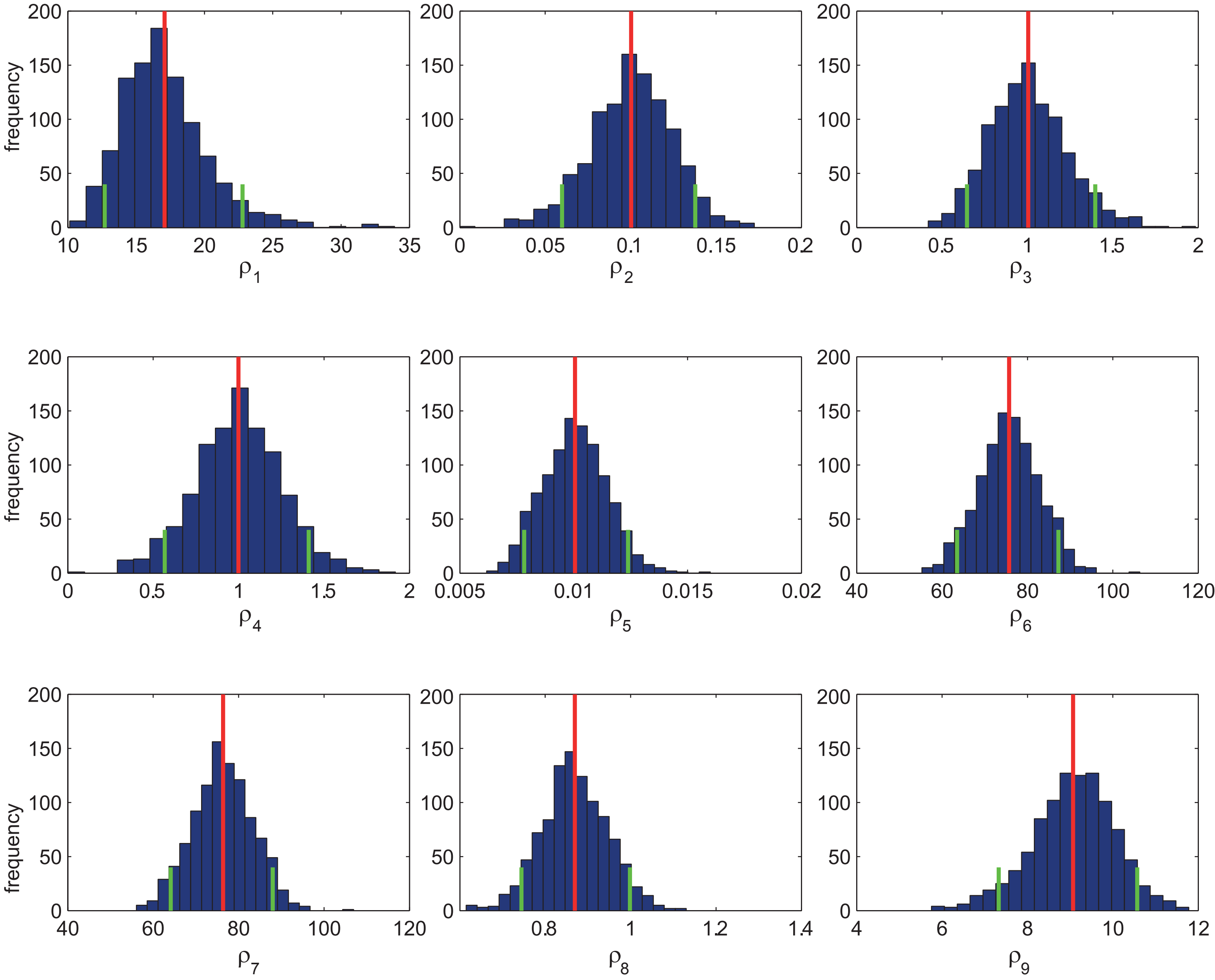

Parameter distribution histograms and Monte Carlo confidence intervals (95 %) for the gene regulation toggle switch. Distribution means are indicated by vertical red lines and confidence intervals are enclosed by green lines.
